# IL-17C expression and its correlation with pediatric adenoids: a preliminary study

**DOI:** 10.7150/ijms.49244

**Published:** 2020-09-20

**Authors:** Hyun Jin Min, Kyung Soo Kim

**Affiliations:** Department of Otorhinolaryngology-Head and Neck Surgery, Chung-Ang University College of Medicine, Seoul, Republic of Korea.

**Keywords:** adenoids, IL-17C, IL-17RE, Hsp70, innate immunity

## Abstract

**Objective:** Interleukin-17 (IL-17) C is a cytokine expressed by epithelial cells in response to bacterial stimulation. In contrast to other members of the IL-17 family of cytokines, IL-17C is upregulated early during infection, maintains integrity of the epithelial layer barrier, and mediates the innate immune response. We investigated the expression profile of IL-17C in pediatric adenoids.

**Methods:** Pediatric adenoid tissues and lavage fluids were collected from a total of 38 subjects. The *Limulus* amebocyte lysate test and real-time PCR using *Staphylococcus aureus* primers were performed to evaluate bacterial contents in adenoids. Expression of IL-17RE in adenoids was analyzed using real-time polymerase chain reaction and western blot. The expression of IL-17C was evaluated by western blot and immunohistochemistry and compared between allergic rhinitis (AR) and control subjects. The levels of Hsp27, Hsp70, and IL-17C in adenoid lavage fluids were evaluated by enzyme-linked immunosorbent assay, and the correlation between these molecules was statistically analyzed.

**Results:** The pediatric adenoids were found to be exposed to bacteria and had a normal flora comprising both gram-negative and -positive bacteria. IL-17RE, an IL-17C specific receptor, was highly expressed in the epithelium of adenoids. IL-17C was expressed in all evaluated adenoid tissue samples, irrespective of the allergic status of the patient. IL-17C secretion was detected in half of the adenoid lavage fluid samples and was associated with Hsp70 level.

**Conclusion:** Our findings indicate the possible role of pediatric adenoids in innate immunity modulation via an innate immunity-associated cytokine.

## Introduction

Nasopharyngeal adenoids are continuously exposed to both airborne and gastrointestinal antigens as they are anatomically located between the upper respiratory and digestive tracts 1. The adenoids are composed of nasopharyngeal-associated lymphoreticular tissues and are a major part of the Waldeyer's lymphoid ring in humans. Histologically, adenoids have pseudostratified columnar epithelium and subepithelial lymphoid tissue. This tissue is separated by connective tissue septa to form lymphoid follicles. Lymphoid follicles contain immature and mature lymphocytes, and are involved in the secretion of immunoglobulins, such as IgA, thereby forming a part of the adaptive immunity 2. These anatomical and histological characteristics of adenoids are known to play a role in host defense against invading pathogens 3,4. Furthermore, it was recently demonstrated that the epithelium of adenoids expresses tight junction proteins and Toll-like receptors (TLRs), and likely plays a role in the initiation of innate immunity 5.

The interleukin-17 (IL-17) family of cytokines plays a key role in various inflammatory conditions. The family has 6 subtypes of IL-17 (A-F) with molecular masses ranging from 20 to 30 kDa and lengths of 163-202 amino acids 6. Studies have demonstrated that these cytokines have diverse inflammatory effects on a broad range of cellular targets, including epithelial and endothelial cells, fibroblasts, osteoblasts, and macrophages 7. IL-17F exhibits the highest homology with IL-17A and is expressed by activated T cells in response to IL-23 stimulation and promotes inflammatory responses 8. IL-17D is expressed in resting CD4 T cells and stimulates the production of IL-6, IL-8, and granulocyte-macrophage colony-stimulating factor in endothelial cells 9. IL-17C is expressed in epithelial cells of upper and lower respiratory organs and secreted extracellularly to function as an immunological mediator in innate immunity 12. A study demonstrated that polyinosinic-polycytidylic acid (polypI:C) induces IL-17C expression through TLR-3, and that both polypI:C and IL-17C increase the expression of antimicrobial peptides, which are important mediators of innate immune response in human bronchial epithelial cells 10. Another study reported the expression and function of IL-17C in nasal epithelium and its role in the pathogenesis of chronic rhinosinusitis with nasal polyposis 11. In contrast, not much is known about IL-17B.

We have previously shown that human adenoid tissues express heat shock protein (Hsp)27 and Hsp70 and suggested that the adenoid epithelium plays a role in innate immune response 13. Therefore, we hypothesized that adenoids may also express IL-17C, which is an active modulator of innate immunity. In this study, we evaluated the expression levels of IL-17C in adenoids and assessed the role of human adenoids in innate immunity.

## Materials and Methods

### Study participants

This study was approved by the Institutional Review Board of Chung-Ang University College of Medicine, and informed consent was obtained from all enrolled participants. All experiments were performed in accordance with the approved guidelines and regulations, and the experimental protocols were approved by the institutional review boards of Chung-Ang University College of Medicine. Thirty-eight children (21 boys and 17 girls; mean age, 5.03 years; range, 3-7 years) were enrolled in this study (Table 1). Enrolled patients underwent adenotonsillectomy for upper airway obstruction and sleep disorders between July 2015 and March 2016 at the Department of Otorhinolaryngology, Chung-Ang University Hospital (Seoul, Korea). Ear, nose, and throat examination, including nasal endoscopy, and paranasal sinus X-rays, was performed as part of the preoperative routine evaluation. OSA-18 assesses the quality of life and disordered breathing and discomfort in pediatric sleep; the OSA-18 score was obtained for all enrolled subjects the day before surgery 14. Ten of the subjects were further evaluated for the presence of allergic rhinitis (AR), through a skin prick test (SPT) and were divided into AR and control groups. Five patients were diagnosed with AR as they fulfilled both the following criteria: i) presented persistent rhinitis symptoms (more than four days per week and for more than four weeks) for over one year, as per the Allergic Rhinitis and its Impact on Asthma (ARIA) guidelines 15, and ii) tested positive for SPT (wheal diameter ≥ 3 mm more than the negative control at 15 min) to at least one allergen. The rest of the patients (N = 5) tested negative for SPT and had no nasal and allergic symptoms and were placed in the control group. None of the subjects had congenital anomalies or systemic diseases such as asthma. Those who took any medication 2 weeks prior to the surgery were excluded from this study.

### Adenoid tissue and lavage fluid collection

Adenoid secretions were collected using a protocol previously established by us, with minor modifications 16. Briefly, normal saline solution (5 mL) was inserted into the surface of the adenoid through a 30° endoscope and approximately 3 mL of the sample was retrieved after 2 s. The adenoid secretion sample was immediately centrifuged and the clear supernatant fraction was used for further experiments. The amount of proteins in lavage fluid was estimated using the bicinchoninic acid assay (Thermo Fisher Scientific, Waltham, MA, USA). After obtaining adenoid lavage fluid samples, adenoid tissues (approximately 0.5×0.5 cm) were harvested using adenoid cutting forceps before adenoidectomy. All samples were immediately stored at -70°C.

### Limulus amebocyte lysate test

The *Limulus* amebocyte lysate (LAL) test (Lonza, Basel, Switzerland) was performed for 8 adenoid tissues to evaluate the endotoxin levels within the tissues. The presence of endotoxin in the tissue is an indicator of the presence of endotoxin-secreting gram-negative bacteria. Briefly, a sample was dispensed in an endotoxin-free tube and then 50 μL of LAL, 100 μL of subtraction solution, and 100 μL stop reagent were serially added as per the manufacturer's protocol. The absorbance of each tube was measured at 405 nm. Normal saline was used as negative control.

### Quantification of bacterial colony-forming units

To quantify the number of bacterial cells in each adenoid tissue sample, we constructed a standard curve of bacterial colony-forming units using cycle threshold (Ct) values. For this, we cultivated *Staphylococcus aureus* in 1/10 serial dilutions on bacterial culture media plates, and counted the colony-forming units. Real-time polymerase chain reaction (PCR) was performed with the extracted *S. aureus* genomic DNA using the 16s rRNA primers: 27F (5'-GAG TTT GAT CCT GGC TCA G-3'); 518R (5'-ATT ACC GCG GCT GCT GG-3') to measure Ct values and thereby estimate the number of bacterial colony-forming units. The real-time PCR was performed using the TaqMan Universal PCR Master Mix (Applied Biosystems, Waltham, MA, USA). The standard curve depicting the Ct values for the corresponding colony-forming units was constructed based on these experiments. After constructing the standard curve, genomic DNA was isolated from adenoid tissues (N = 18) using a QIAamp DNA Mini Kit (QIAGEN, Venlo, Netherlands), according to the manufacturer's protocol. Next, we performed real-time PCR using the 16s rRNA primer pair with the DNA extracted from each adenoid tissue sample and obtained the Ct values. Using the standard curve, the total number of bacterial colony-forming units was calculated from the Ct values.

### Real-time PCR

Three randomly selected adenoid tissues were used for detection of IL-17 receptor (IL-17RE) mRNA expression. Total RNA was isolated using TRIzol (Invitrogen Life Sciences, Grand Island, NY, USA) and cDNA was synthesized using HighCapacity cDNA Reverse Transcription Kits (Applied Biosystems) with random hexamer primers (PerkinElmer Life Sciences, Santa Clara, CA, USA) 17. TaqMan® universal PCR master mix (Thermo Fisher Scientific) was used for real-time PCR and target-specific probes were constructed. The primers sequences used were as follows: IL-17RE forward primer, 5'-TCCTGGAATGTAAGCATGGATACC-3', IL-17RE reverse primer, 5'-GGAAGGGAATGATGAGGTCTAGTG-3'; and glyceraldehyde 3-phosphate dehydrogenase (GAPDH) forward primer, 5'-AAGGTCATCCCTGAGCTGAA-3', GAPDH reverse primer, 5'-TGCTGTAGCCAAATTCGTTG-3'.

### Western blot assay

Tissues were lysed using radioimmunoprecipitation assay (RIPA) lysis buffer containing a mixture of protease inhibitors (Sigma-Aldrich, St. Louis, MO, USA). Adenoid protein samples (30 μg) were separated by 10% sodium dodecyl sulfate polyacrylamide gel electrophoresis and transferred to a nitrocellulose membrane. The membrane was first incubated with primary antibodies against IL-17C and GAPDH (Abcam, Cambridge, UK), washed, and then incubated with secondary antibodies, namely horseradish peroxidase-labeled goat anti-rabbit or goat anti-mouse immunoglobulin (Jackson Labs, Bar Harbor, ME, USA). Signals were detected using the Enhanced Chemiluminescence detection reagent (Amersham, Buckinghamshire, UK) and relative band intensities were calculated using the Image J software (National Institutes of Health, Bethesda, MD, USA). The total number of adenoid tissues analyzed in each western blot assay is indicated in the corresponding figure legend.

### Quantification of IL-17C, Hsp27, and 70 in adenoid lavage fluid samples

A total of 38 adenoid lavage fluid samples were evaluated to determine the expression of IL-17C, Hsp27, Hsp70, and *high mobility group box 1* (HMGB1). The amount of IL-17C, Hsp27, and Hsp70 secreted in adenoid lavage fluids was determined using a commercial enzyme-linked immunosorbent assay (ELISA) kit (R&D Systems, Minneapolis, MN, USA), according to the manufacturer's instructions.

### Immunohistochemistry analysis

Three and 10 (5 each from the AR and control groups) adenoid tissues were evaluated in the immunohistochemistry assay for IL-17RE expression and IL-17C expression, respectively. Formalin-fixed, paraffin-embedded adenoid section slides were prepared. For antigen retrieval, slides were covered with sodium citrate buffer solution (pH 6.0; 0.01 M) for 10 min. After blocking nonspecific binding sites, the sections were incubated overnight with the primary antibodies against IL-17RE and IL-17C (Abcam) at 4°C. Secondary antibodies were then applied using the Dako EnVision+ kit (Dako, Carpinteria, CA, USA) for 30 min. Then, the sections were further incubated at room temperature for 20 min and stained with 3,3'-diaminobenzidine for 10 min. Secondary antibody only was used as negative control.

### Measurement of adenoid size

We evaluated the adenoid size by two measurement ratios, as previously described 18, 19. The distance between the outermost point of convexity in the adenoid shadow and the sphenobasiocciput was divided by the distance between the sphenobasiocciput and the posterior end of the hard palate, and named as the adenoid-nasopharynx (A/N) ratio 18. The airway distance (the width of the airway at the narrowest point owing to the enlarged adenoid) was divided by the thickness of the soft palate (soft palate size), and it was named as adenoid-soft palate (A/S) ratio 19.

### Statistical analyses

Data are presented as means ± standard deviations. The level of IL-17C, Hsp27, and Hsp70 between two groups was compared using the Mann-Whitney U test. Univariate and multivariate analyses were performed to evaluate the relationship between the level of IL-17C and clinical characteristics of the patients. *P*-values < 0.05 were considered statistically significant. The SPSS Statistics 18 (IBM Corp, Armonk, NY, USA) software package was used to perform all statistical analyses.

## Results

### Pediatric adenoids contain gram-negative and -positive bacteria

**Figure 1:**
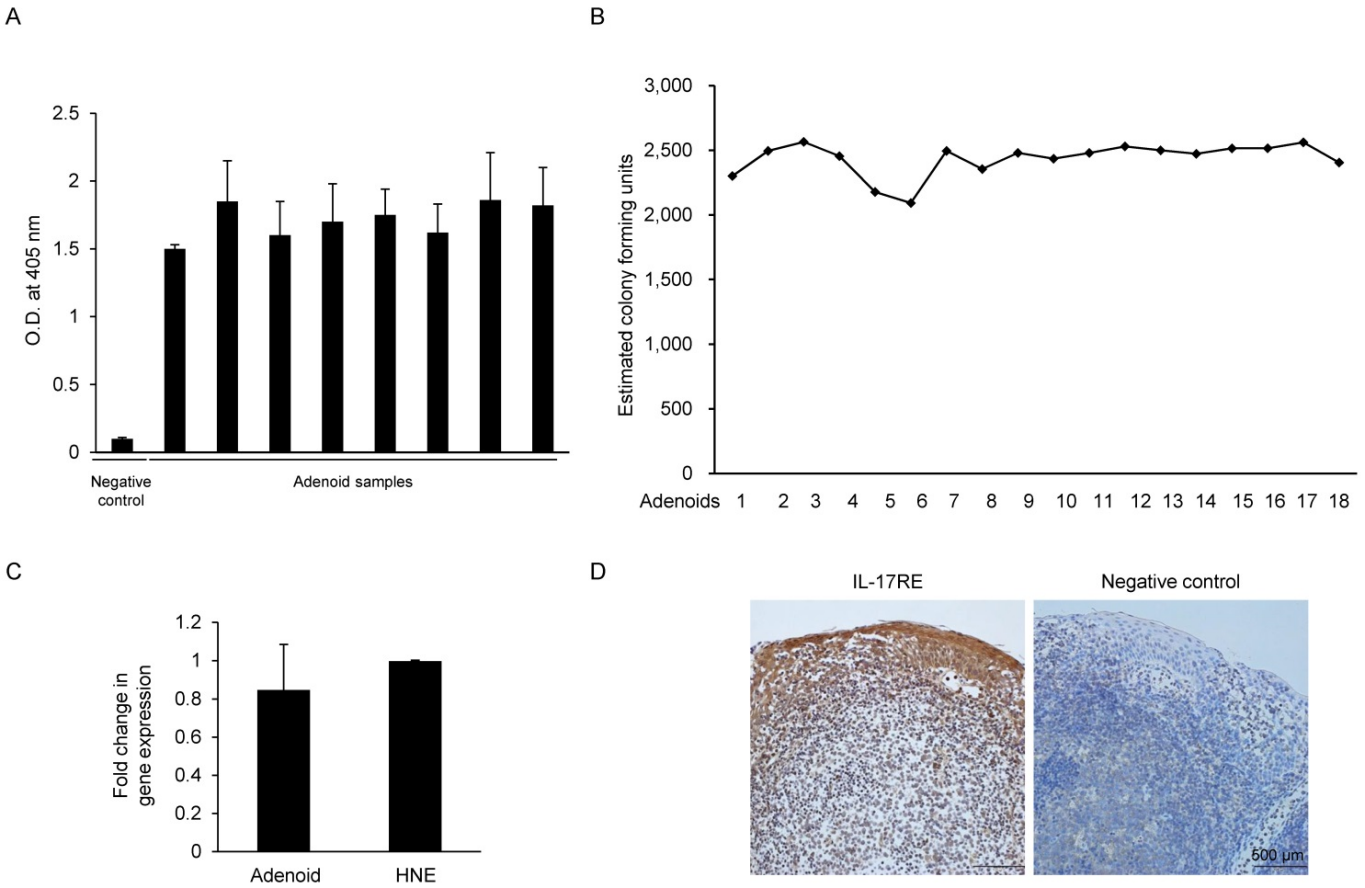
Estimation of the endotoxin level, total bacterial count, and expression of IL-17RE in adenoids from pediatric patients. (A) The endotoxin level in each adenoid sample was measured using the *Limulus* amebocyte lysate test. Saline solution was used as a negative control. (N = 8). (B) The total bacterial count in the adenoid tissue was estimated using a standard curve plotted using *S. aureus* primers. Real-time PCR was performed using adenoid tissue, and the cycle threshold (Ct) value was obtained. The Ct value was plotted on the standard curve, and the total bacterial count was inversely calculated (N = 18). (C) Gene expression of IL-17RE relative to GAPDH expression was evaluated by a real-time PCR assay (N = 3). (D) Immunohistochemistry analysis of IL-17RE in adenoid tissues. (N = 3). Abbreviations: O.D.: optical density; HNE, N: number of samples of adenoid tissue used.

Since IL-17C is expressed in response to bacterial stimulation, we examined whether the pediatric adenoid tissues used in our experiments were exposed to various bacterial stimulations. We also evaluated the type and total number of bacteria in each adenoid tissue sample, and we assayed a total of 26 adenoid tissue samples using the LAL test and real-time PCR. The water used in the sample to construct the standard curve was used as negative control. The LAL test results showed that all the randomly chosen adenoid samples (N = 8) contained endotoxin, suggesting continuous exposure to gram-negative bacteria (Fig. 1A). Next, we performed real-time PCR of adenoid-derived DNA samples (N = 18) using the Ct values and inversely calculated the number of total bacterial colony-forming units from the standard curve (Fig. 1B). We found that adenoids used in our experiments contained substantial amounts of *S. aureus*. Our results suggest that adenoid samples contained both gram-negative and -positive bacteria, but could not find individual differences between the adenoid tissues.

### Pediatric adenoids express IL-17RE, an IL-17C specific receptor subunit

IL-17C signaling requires a heterodimeric complex of IL-17RE and IL-17RA. In contrast to IL-17RA, which is ubiquitously expressed, IL-17RE is expressed primarily by epithelial cells, making these cells responsive to IL-17C. We hypothesized that if human adenoid epithelial cells express IL-17C, they should also express the specific receptor of the cytokine, IL-17RE. Real-time PCR performed using the total RNA extracted from pediatric adenoid tissues (N = 3) with IL-17RE specific-primers confirmed the presence of IL-17RE mRNA. The level of IL-17RE expression in adenoid epithelial cells was similar to that in upper airway epithelial cells, which were used as a positive control (Fig. 1C). Immunohistochemical staining of the adenoid epithelial cells using IL-17RE-specific antibodies was positive, further confirming that pediatric adenoids express IL-17RE (Fig. 1D).

### IL-17C expression is not different between AR and control groups

**Figure 2:**
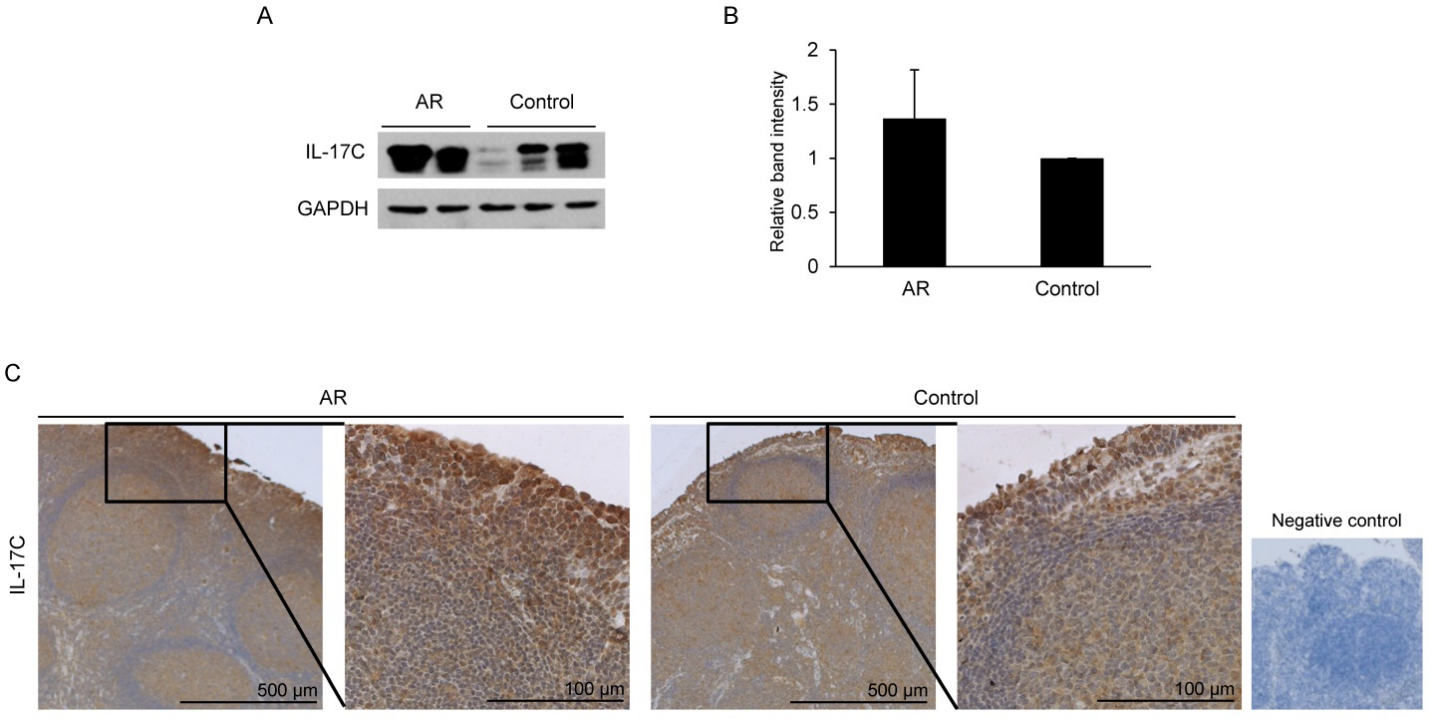
Evaluation of IL-17C expression in adenoid tissues and comparison of its levels in allergic rhinitis (AR) and control groups. (A) Western blot assay was performed to evaluate the expression of IL-17C in both AR (N = 5) and control patients (N = 5). (B) Relative band intensity was calculated and compared using Image J software (N = 10). (C) Same adenoid tissues were used for the immunohistochemistry assay.

Western blot and immunohistochemistry analysis were performed to assess the expression of IL-17C in adenoids. A total of 10 adenoid tissue samples were evaluated, and they were grouped as AR and control based on nasal and allergic symptoms and SPT. Western blot analysis revealed the constitutive expression of IL-17C in all adenoid samples (Fig. 2A). Furthermore, the difference in the levels of IL-17C expression in AR and control groups was not statistically significant (Fig. 2B).

Next, we collected pediatric adenoid lavage fluids to analyze the presence of IL-17C in the extracellular fluid of pediatric adenoids. In total, 38 adenoid fluid samples were harvested and used for ELISA (Table 1). We also assessed the presence of Hsp27 and Hsp70 in adenoid lavage fluids, as suggested by an earlier study 13. IL-17C was detected in 47% of adenoid lavage fluids (18 of 38 samples), and the mean concentration was 1749.98 pg/mL. Hsp27 (mean, 773.89 ± 442.51 pg/mL) and Hsp70 (mean, 10165.64 ± 4378.93 pg/mL) were detected in all adenoid fluids (Table 1).

SD: standard deviation; Hsp: heat shock protein; HMGB1: high mobility group box 1; OSA score: obstructive sleep apnea score; A/N: adenoid-nasopharynx; A/S: adenoid-soft palate.

### IL-17C levels in adenoid lavage fluids is associated with Hsp70 expression

**Figure 3:**
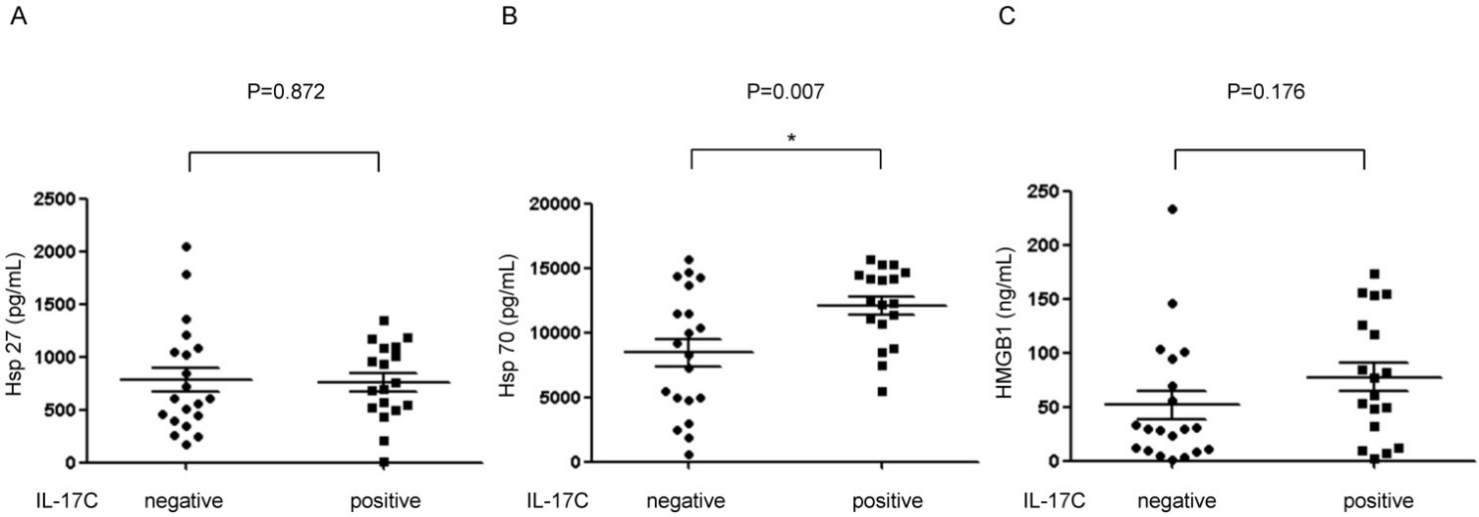
Detection of IL-17C in adenoid lavage fluid samples and correlation with damage-associated molecular patterns (DAMPs). The secreted amounts of (A) Hsp27, (B) Hsp70, and (C) HMGB1 in adenoid lavage fluid samples were evaluated using ELISA. The level of these molecules was statistically analyzed between IL-17C positive and negative adenoid lavage fluid samples. *P* < 0.05 was regarded as statistically significant.

As Hsps and IL-17C are all important mediators of innate immunity in epithelial cells, we examined the correlation between these molecules using ELISA. We found that the level of Hsp70 was higher in IL-17C positive adenoid lavage fluids than in the IL-17C negative fluids (Fig. 3). Further, univariate and multivariate linear regression analyses also revealed that Hsp70 was the only factor that was directly associated with the level of IL-17C (Table 2). Interestingly, no correlation was found between the expression levels of Hsp27 and IL-17C.

## Discussion

Human adenoids are composed of epithelial cells and are germinal centers of lymphocytes. Traditionally, the main function of adenoids is considered to be production of secretory IgA in germinal centers. However, recent findings suggest that adenoids may play an important role in innate immunity as well. Epithelial tight junction barrier of human adenoid epithelium helps maintain the epithelial integrity and plays a crucial role in the initial defense against pathogens 5. Adenoid epithelial cells express TLRs in their membrane 20. We have previously reported that adenoids also express damage-associated molecular patterns (DAMPs) which, upon stimulation, are secreted extracellularly and are, therefore, present in adenoid lavage fluid 13. In this study, we show that IL-17C is expressed in pediatric adenoids and that the expression level of this cytokine is associated with the level of Hsp70 in adenoid lavage fluids. Our findings further support the active role of adenoids in the initiation of innate immunity.

Recent studies demonstrate that IL-17C plays a key role in regulating the innate immune function of epithelial cells and acts as a link between inflammation and maintenance of the mucosal barrier function during infection 11. IL-17C is induced in mucosal and cutaneous epithelial cells in response to bacterial stimulation and in epidermal keratinocytes by bacterial and pathogen-associated molecular patterns (PAMPs) 21. Bacterial infection or stimulation with PAMPs activates the TLR signaling pathway, which is important in the initiation of innate immunity, suggesting that IL-17C may be important to innate immunity 21. As we are interested in evaluating the function of adenoids in host innate immunity, we focused on the expression profile of IL-17C. High and equal expression levels of IL-17C were found in the epithelium of 10 randomly selected adenoids. IL-17C is functionally unique from the rest of the members of the IL-17 family because it is induced by various stimulations, such as bacterial infection and cytokine treatment. This suggested that Th2 cytokines and a Th2-dominant inflammatory condition may influence the expression of IL-17C. Furthermore, there was a report that the regional levels of IL-17C were higher in the nasal mucosa of allergy and asthma subjects than in that of control subjects 22. However, our results showed no significant difference in the expression level of IL-17C between AR and control groups. We presume that other unknown factors may be involved in regulating the expression level of IL-17C in adenoid tissues.

Interestingly, about half of the adenoid lavage fluid samples contained extracellularly secreted IL-17C; therefore, we looked into some possible factors that regulate IL-17C secretion. Since human adenoids also secrete DAMPs, such as Hsp27 and Hsp70 13, we evaluated the expression levels of DAMPs and their association with IL-17C. Similar to our previous findings, Hsp27 and Hsp70 were detected in all adenoid lavage fluid samples. We also found that the Hsp70 level was higher in adenoid lavage fluid samples that also contained IL-17C secretions and that the association was statistically significant. However, Hsp27 expression was not found to be associated with the IL-17C level. This was an interesting finding because IL-17C and Hsp70 have similar characteristics. Both have pro- and anti- inflammatory functions in the extracellular space of adenoids. The cytokine exhibits a protective role in dextran sulfate sodium induced-colitis and displays a pro-inflammatory function in psoriasis mouse model 21. Similarly, extracellular Hsp70 delivers anti-inflammatory and pro-inflammatory signals through stimulation by Siglec-5 and -14, respectively 23. Furthermore, the function of Hsp70 is affected by its extracellular concentration 24. We conclude that extracellular Hsp70 and IL-17C may be regulated by similar stimulations and signaling pathways and exert pro- and anti-inflammatory responses.

Stimulation of IL-17C is insufficient to induce secretion of IL-6, but co-stimulation with *Pseudomonas aeruginosa* leads to synergistic activation of human bronchial epithelial cells to release IL-6 25. IL-17C also functions synergistically with tumor necrosis factor-α and IL-1β in inducing β-defensin-2 from keratinocytes 21. These findings suggest that secreted IL-17C may exhibit synergistic effects with other cytokines or bacterial co-infection in adenoid tissues. Among the IL-17 family of cytokines, IL-17C is the only member that is induced in human respiratory epithelial cells by bacterial stimuli and specific ligands 25. This prompted us to explore the possibility that bacterial infection may regulate IL-17C secretion in adenoids. Bacterial loads were found to be similar in all adenoids among the various samples tested. We are further investigating the effect of the microbiome on the secretion of IL-17C. It will also be interesting to explore the possible synergistic roles of Hsp70 and IL-17C in modulating the innate immune system.

The mean Hsp70 level (10165.64 ± 4378.93 pg/mL) measured in this study in the adenoid lavage fluid was found to be significantly higher than the previously reported Hsp70 level (880 pg/mL (270-1880 pg/mL)) in sputum samples 26. Our ELISA-measured IL-17C levels (1749.98 ± 1353.07 pg/mL) in adenoid lavage fluid were also found to be higher than the previously reported IL-17C levels (< 400 pg/mL) in lower airway epithelial cell secretory fluids 25. The higher levels of Hsp70 and IL-17C in the extracellular space of adenoids than in the lower airways may indicate a more significant presence of innate immune regulatory factors in the nasopharyngeal airway. So far, the expression of IL-17C in the nasal cavity has been demonstrated only through western blot analysis 11. Therefore, an absolute quantification of IL-17C levels in the upper airway and its comparison with the expression levels of IL-17C in the nasopharyngeal and lower airways will be interesting to explore.

IL-17C is preferentially expressed in epithelial cells but not in fibroblasts and peripheral blood monocyte cell cultures. Similarly, IL-17RE is also expressed preferentially in epithelial cells 21. Using real-time PCR and immunohistochemistry, we showed that adenoid epithelial cells expressed IL-17RE. Further, the rapid induction of IL-17C expression after bacterial exposure suggests that the cytokine functions in an autocrine manner in epithelial cells 21. Along these lines, we assume that IL-17C may activate adenoid epithelial cells in an autocrine manner and augment the innate immune response.

This study has several limitations. First, since the number of patients enrolled in this study is limited, our findings are preliminary; therefore, a similar study with a large sample size is required for the confirmation of our hypothesis. Second, our study could not identify the exact cellular origin of IL-17C production. Adenoid tissues are composed of both epithelium and germinal centers of lymphocytes. Therefore, performing similar experiments using only the epithelial cells of adenoids may confirm our preliminary findings that adenoid epithelial cells possibly express and secrete IL-17C. Third, an in-depth evaluation of the downstream signaling pathways and immunological function of IL-17C is required for a complete understanding of the immunological significance of IL-17C in adenoids.

## Conclusion

In conclusion, pediatric human adenoids express IL-17C, which is an important inflammatory mediator of innate immunity. Since IL-17RE is constitutively expressed in the adenoid epithelium, there is a possibility that adenoids respond to IL-17C in an autocrine manner. Adenoids secrete IL-17C extracellularly, the levels of which are significantly associated with the levels of Hsp70. These findings suggest that human adenoids not only play an important role in adaptive immunity as an antibody-secreting organ but also possibly play a role in mediating innate immunity.

## Figures and Tables

**Table 1:** Clinical characteristics of the participants (N = 38)

Characteristic	Value
**Sex**	
Female	17
Male	21
**Age, years ± SD (range)**	5.03 ± 1.05 (3-7)
**Hsp 27, pg/mL (N = 38)**	773.89 ± 442.51
**Hsp 70, pg/mL (N = 38)**	10165.64 ± 4378.93
**HMGB1, ng/mL (N = 38)**	63.98 ± 58.78
**IL-17C, pg/mL (N = 18)**	1749.98 ± 1353.07
**OSA score (range)**	61.66 ± 20.62 (31-117)
**A/N ratio ± SD**	0.72 ± 0.11 (0.47-0.90)
**A/S ratio ± SD**	0.46 ± 0.16 (0.22-0.92)

**Table 2:** Univariate and multivariate linear regression analyses to identify factors associated with IL-17C

Variables for IL-17C	Univariate		Multivariate	
	Β (SE)	*P*-value	Β (SE)	*P*-value
Age	-0.145 (0.315)	0.646	-0.075 (0.421)	0.859
Hsp 27	0.000 (0.001)	0.868	0.000 (0.001)	0.769
Hsp 70	0.000 (0.000)	0.015	0.000 (0.000)	0.026
HMGB1	0.008 (0.006)	0.178	-0.008 (0.010)	0.445
OSA score	-0.005 (0.016)	0.764	-0.006 (0.064)	0.800
A/N ratio	0.840 (2.951)	0.776	-0.564 (5.674)	0.921
A/S ratio	0.788 (2.043)	0.700	1.892 (3.829)	0.621

Hsp: heat shock protein; HMGB1: high mobility group box 1; OSA score: obstructive sleep apnea score; A/N: adenoid-nasopharynx; A/S: adenoid-soft palate.
